# Non-invasive, multimodal analysis of cortical activity, blood volume and neurovascular coupling in infantile spasms using EEG-fNIRS monitoring

**DOI:** 10.1016/j.nicl.2017.05.004

**Published:** 2017-05-13

**Authors:** Emilie Bourel-Ponchel, Mahdi Mahmoudzadeh, Aline Delignières, Patrick Berquin, Fabrice Wallois

**Affiliations:** a INSERM U 1105, GRAMFC, CURS, Centre Hospitalier Universitaire d'Amiens, F-80054 Amiens, France; b Service d'explorations fonctionnelles du système nerveux pédiatrique, Centre Hospitalier Universitaire d'Amiens, F-80054 Amiens, France; c Unité de neurologie pédiatrique, Centre Hospitalier Universitaire d'Amiens, F-80054 Amiens, France

**Keywords:** EEG, electroencephalogram/electroencephalography, EMG, electromyography, fNIRS, functional near infrared spectroscopy, HbO, oxyhemoglobin, Hb, deoxyhemoglobin, HbT, total hemoglobin, HRF, hemodynamic response function, NVC, neurovascular coupling, PET, positron emission tomography, SPECT, Single photon emission computed tomography, TFR, time frequency representation, Infantile spasm, Neurovascular coupling, Cerebral blood volume, Electroencephalography, Optical imaging

## Abstract

Although infantile spasms can be caused by a variety of etiologies, the clinical features are stereotypical. The neuronal and vascular mechanisms that contribute to the emergence of infantile spasms are not well understood. We performed a multimodal study by simultaneously recording electroencephalogram and functional Near-infrared spectroscopy in an intentionally heterogeneous population of six children with spasms in clusters. Regardless of the etiology, spasms were accompanied by two phases of hemodynamic changes; an initial change in the cerebral blood volume (simultaneously with each spasm) followed by a neurovascular coupling in all children except for the one with a large porencephalic cyst. Changes in cerebral blood volume, like the neurovascular coupling, occurred over frontal areas in all patients regardless of any brain damage suggesting a diffuse hemodynamic cortical response. The simultaneous motor activation and changes in cerebral blood volume might result from the involvement of the brainstem. The inconstant neurovascular coupling phase suggests a diffuse activation of the brain likely resulting too from the brainstem involvement that might trigger diffuse changes in cortical excitability.

## Highlights

• Diffuse CBV changes and motor contraction occurred simultaneously during spasms.
• Initial CBV changes suggest an initial involvement of the brainstem.
• CBV is followed by an inconstant NVC reflecting a diffuse cortical activation.
• Complex interplays between the cortico-sub-cortical structures characterize spasms.
• EEG-fNIRS can study interplay between cortico-sub cortical structures in epilepsy.

## 1 Introduction

Infantile epileptic spasms (described for the first time by W.J. West in 1841) constitute the most frequent form of epileptic encephalopathy (West, 1841). The spasms typically start during the first year of life, with an incidence estimated at between 0.25 and 0.42 per 1000 live births (Mackay et al., 2004, Lux, 2013, Go et al., 2012, Pavone et al., 2014). Clinically, spasms are observed as contractions of the neck, trunk and limb flexor and/or extensor muscles that last for one or 2 s and typically occur in clusters upon awakening or before sleep (Kossoff, 2010). On an electroencephalogram (EEG), a spasm is usually associated with a widespread, high-voltage slow wave and then an attenuation of the background activity that lasts for a second or two (Fusco et al., 1993, Kellaway et al., 1979).

The pathophysiology of infantile spasms is not well understood. Although spasms can be ascribed to a variety of etiologies (such as perinatal events, brain malformations, and genetic or chromosomal abnormalities), the clinical and electroencephalographic phenotypes are stereotypical - suggesting the involvement of highly specific, age-dependent processes that involve both cortical and subcortical structures (Lado and Moshé, 2002). In positron emission tomography (PET) and single-photon emission computed tomography (SPECT), hemodynamic and metabolic changes have been described within cortical malformations (Hrachovy and Frost, 1989, Chiron et al., 1993, Chugani et al., 1990, Haginoya et al., 2013), but also in remote cortical and subcortical structures, including the brainstem (Hrachovy and Frost, 1989, Chiron et al., 1993, Chugani et al., 1990, Chugani et al., 1992, Haginoya et al., 1998, Haginoya et al., 1999, Haginoya et al., 2013). Some authors suggested that the involvement of the cortex is restricted to the cortical malformations (i.e. cortical dysplasia), which are likely to trigger neuronal networks in the brainstem, which in turn generate a spasms' motor program (Chugani et al., 1992). However, diffuse cortical activation could be also expected from the diffuse high voltage slow waves followed by a diffuse attenuation of background activity and fast rhythms observed on EEG. Finally, symmetric infantile spasms have been reported in patients with extensive cortical lesions (Haginoya et al., 1999) and in a patient with hydranencephaly (Neuville, 1972); suggesting that involvement of the neocortex is not a prerequisite for spasm generation.

To better understand the pathophysiological mechanisms related to each spasm in a series, a multimodal approach with a high time resolution is mandatory.

PET and SPECT, given their low temporal resolution, are inappropriate to evaluate specifically the underlying neurovascular changes related to each spasm in a series. fNIRS has the advantage to monitor fast hemodynamic changes with a temporal resolution of a tenth of a millisecond. In addition fNIRS allows discrimination between changes in blood volume related to vascular regulation and the occurrence of a neurovascular coupling (Buxton and Frank, 1997, Buxton et al., 2004), related to a neuronal activation, regardless of the type of neurons being activated or their orientation. Also, fNIRS will characterize the time course of the successive hemodynamic events as described in epileptic seizures (Wallois et al., 2009, Wallois et al., 2010). Whatever the etiology, the stereotypical clinical aspect of spasms rely on a common anatomical and functional substratum that likely involves the cortex and the subcortical areas. To evaluate the common cortical blood volume changes and or the neurovascular coupling, patients with spasms with heterogeneous etiologies, but that share common clinical features, were deliberately recruited. To ensure that the hemodynamic changes were related to cortical networks layers, a multidistance fNIRS approach was developed simultaneously to the Video-EEG, EMG recordings (Huber et al., 2010).

## 2 Materials and methods

### 2.1 The study population

Children attending the Pediatric Neurology Unit or the Functional Exploration of the Pediatric Nervous System Unit at Amiens University Medical Center (Amiens, France) were screened for eligibility. Patients aged from 3 months to 3 years, presenting with infantile spasms (flexor contractions) in clusters and meeting the International League against Epilepsy's classification were included in the study (Berg et al., 2010).

### 2.2 Methodology

#### 2.2.1 Data acquisition

##### 2.2.1.1 Collection of clinical data

Each participant's personal information, family history, disease progression and EEG and brain imaging results were recorded. The spasms' clinical characteristics (the type of movement, symmetry, and occurrence in clusters or not) were also noted. Simultaneous EEG and fNIRS recordings were performed at the bedside.

##### 2.2.1.2 Video EEG-EMG

**Fig. 1:**
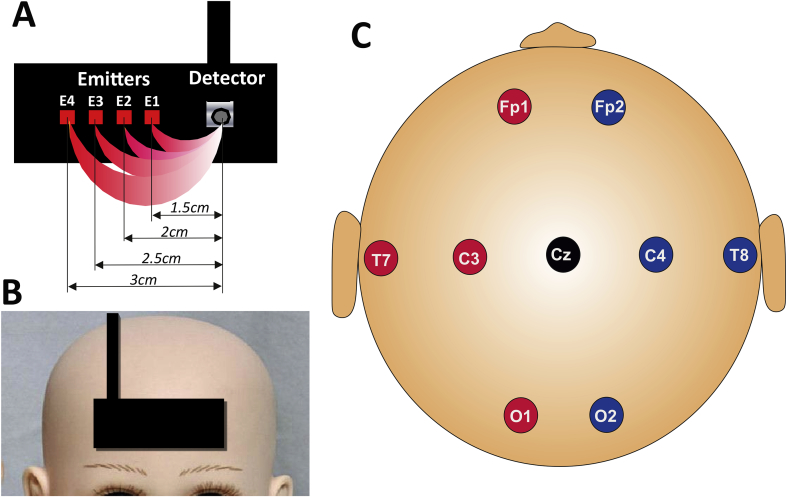
EEG and optical imaging acquisition data A–B: A patch comprising four pairs of optical fibers (one fiber for each wavelength in each pair) and a detector has been positioned in the middle of the forehead. The four channels (A1–A4) differed in the distance between the emitter and the detector (15, 20, 25 and 30 mm for channels A1 (D-E1), A2 (D-E2), A3 (D-E3) and A4 (D-E4), respectively). C: 9 electrodes were disposed according to the 10–20 system, with a frontal reference.

Synchronized video-EEG was performed on a Coherence 3NT® (Deltamed, France) (9 electrodes, disposed according to the 10–20 system, with a frontal reference; sampling rate: 512 Hz) (Fig. 1). Two electromyogram (EMG) electrodes were positioned on the right and left deltoid muscles, respectively. Respiratory activity was monitored with a piezoelectric system. The heart rate was monitored through two skin electrodes positioned on the chest. Impedances were monitored and maintained below 5 kΩ. Data was acquired and stored without any filtering.

##### 2.2.1.3 Optical imaging

fNIRS was performed with a multidistance spectrophotometer operating in the frequency domain (Imagent® ISS Inc., IL, USA). Laser light was transmitted to photodiodes at the scalp surface via optical fibers. The laser's two emission wavelengths (λ = 690 nm and λ = 830 nm) corresponded to the infrared absorption spectra of [HbO] and [Hb], respectively.

A patch comprising four pairs of optical fibers (one fiber for each wavelength in each pair) and a detector was positioned in the middle of the forehead. The four channels (A1–A4) differed in the distance between the emitter and the detector (15, 20, 25 and 30 mm for channels A1, A2, A3 and A4, respectively) (Fig. 1). With regard to the propagation of photons in the different brain structures, this multidistance methodology enables the analysis of hemodynamic changes at various depths below the scalp surface (5, 7, 8.5 and 10 mm for channels A1, A2, A3 and A4, respectively) (Huber et al., 2010). It is generally considered that optical changes related to cortical activity decrease as the emitter-detector distance decreases and, conversely, that optical changes related to superficial, non-cortical hemodynamic perturbations decrease as the distance increases (Patterson et al., 1995).

##### 2.2.1.4 Ethical considerations

The study was approved by the local investigational review board (*CPP Nord-Ouest*, Amiens, France; reference: A00782-39). Prior to inclusion, all parents gave their written, informed consent to their child's participation in the study.

#### 2.2.2 Data processing

EEG and fNIRS recordings were synchronized against an external clock (Mahmoudzadeh et al., 2013).

##### 2.2.2.1 Video EEG-EMG

Video EEG-EMG recordings were analyzed by two experienced neurophysiologists (EB and FW). Spasms were delimited by an abrupt increase in EMG deltoid activities and synchronized video monitoring of the same movement.

In order to determine the onset of EMG activity (T0), a time-frequency representation (TFR), for the frequencies between 4 and 125 Hz, was generated by applying complex demodulation procedures (Hoechstetter et al., 2004, Papp and Harms, 1977) (for details, see Supplemental data).

Video EEG-EMG recordings were then visually inspected, to ensure that no other movements (eye movement, head movement, etc.) preceded the onset of deltoid activation.

EEG data were filtered between 0.5 and 70 Hz. A notch filter was applied and a bipolar montage was used (Deltamed®).

##### 2.2.2.2 Optical imaging

The signal intensities at 690 and 830 nm were converted into relative changes in the [HbO] and [Hb] concentrations by application of the modified Beer-Lambert law. The optical data were band-pass filtered [0.03–0.5 Hz] with a zero-phase filter (Butterworth, order: 6) to eliminate physiological noise (e.g. slow drifts and arterial pulse oscillations). The amplitude of [HbO], [Hb] were normalized. Time series surrounding spasms (T0) were selected. The duration of a time series depended on the time interval between the spasms and the duration of the clinical manifestations but was similar for a given patient and did not change during the cluster of spasms. The time series were analyzed individually. After linear detrending and baseline correction, the segments were averaged for each patient around the reference time T0 (corresponding to the onset of deltoid contractions). The hemodynamic response function (HRF) was determined by simply averaging the selected time series.

To confirm the cortical origin of the hemodynamic changes, we have compared the distance dependent variation of fNIRS signal sets among the 6 patients. We have determined the difference between the maximum and minimum [HbO] values for the four source-detector distances in the period of − 5 to 25 s (T0 is the spasm onset). To facilitate comparisons among different patients, a normalized range values were calculated (Fig. 4B).

## 3 Results

### 3.1 Characteristics of the study population

Between January 2012 and May 2015, 9 patients with infantile spasms were included in the study. In 8 cases, the child was included at the time of diagnosis. In the remaining case, the child (aged 23 months) was included after the observation of recurring spasms in a context of neurotransmitter deficiency and deteriorating general condition. Seven of the 9 patients displayed manifestations during monitoring, and 6 had spasms in clusters. The seventh child had a partial seizure (in the absence of typical spasms) and was thus excluded from the study.

### 3.2 EEG and clinical data

The patients' clinical characteristics, interictal and ictal EEG data are summarized in Table 1 (for more details, see Supplemental data).

**Fig. 2:**
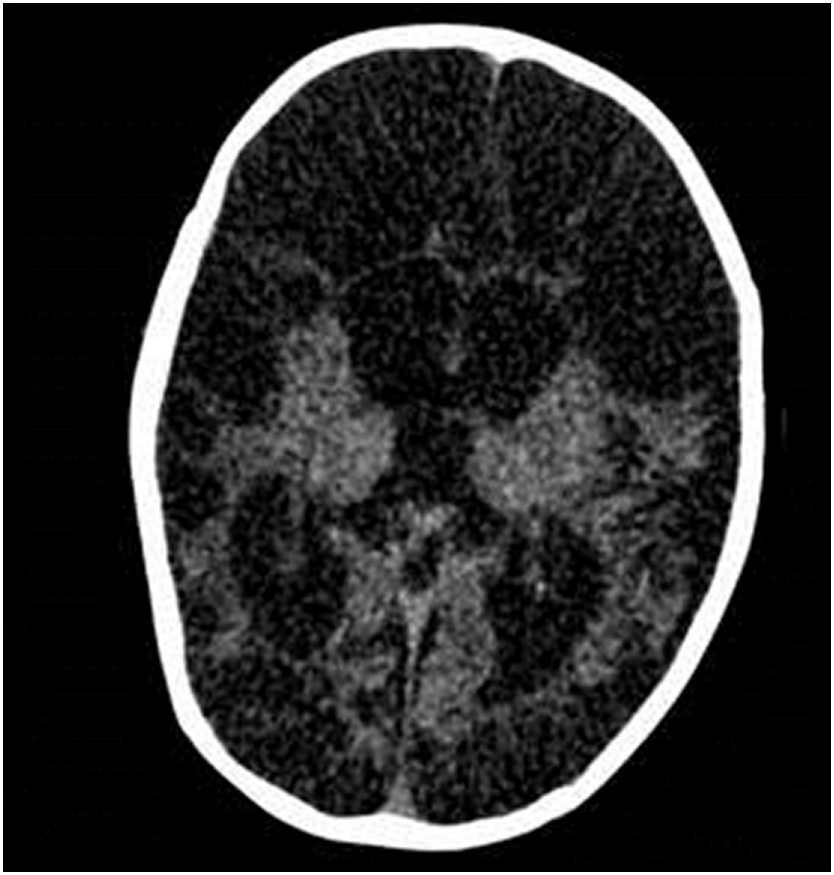
CT scan of patient #6 CT scan of patient #6 found an almost complete destruction of the parenchyma with only persistence of a thin cortical ribbon (resulting in a very large porencephalic cyst).

*Patient #1* presented with idiopathic infantile spasms, beginning at the age of 6 weeks. After 6 months of standard treatment (vigabatrin and hydrocortisone), the patient was spasm free and the clinical outcome was normal. The five other patients presented symptomatic infantile spasms as the result of heterogeneous etiologies. All of them had poor neurological evolution and developmental delay.
*Patient #2* presented symptomatic infantile spasms as the result of left temporo-parietal polymicrogyria.
*Patient #3* was delivered by emergency cesarean section at 40 weeks of gestation, following the observation of acute fetal distress syndrome and perinatal anoxo-ischemia.
*Patient #4* presented with symptomatic infantile spasms as a result of a neurotransmitter deficiency (suggested by a very low level of 5-methyltetrahydrofolate in the cerebrospinal fluid).
*Patient #5* was delivered by cesarean section after 35 weeks of gestation, following the observation of intra-uterine growth retardation. A mitochondrial cytopathy, a metabolic disorder, was diagnosed, with a confirmed lack of complex IV in muscle and liver biopsies. The last patient (*Patient #6*) presented with Group B *Streptococcus* meningoencephalitis at the age of 1 month. The infection had caused severe anoxic and ischemic damage. A CT scan revealed almost complete destruction of the parenchyma and a thin cortical ribbon resulting in a very large porencephalic cyst (Fig. 2).

**Table 1:** Clinical (clinical history, psychomotor development, type of seizures and etiologic diagnostic), interictal and EEG data for patients included in the study.

Patient number	Sex	Perinatal history	Psychomotor developement before spasms	Age at onset spasms	Others seizures	Neuroimaging	Etiologic diagnostic	Number of spasms recording in NIRS-EEG	Mean duration of each pasm	Interval between spasms (second) (min-max)	Interictal EEG	Ictal EEG
1	M	Birth by cesarean at term with acute asphyxia (apgar 6–8-8)	Normal	6 weeks	No	Normal	Idiopathic infantile spasms	6	1–2 s	56 (25–110)	Bilateral high amplitude posterior delta wave	Inconsistent diffuse theta activity before spasms
2	M	Eutocic delivery at term	Normal	5 months	Partial clonic seizure	Left temporo-parietal polymicrogyria	Infantile spasms symptomatic of left cortical malformation	14	1–2 s	10 (7–39)	Asymetrical hypsarythmia with theta slow wave predomintantly in the left hemisphere	Symetrical high amplitude slow wave and flattening of the ongoing activities
3	F	Birth by cesarean at term wirh acute aspyxia (apgar 1–1-2)	Abnormal	5 months	No	Diffuse anoxo-ischemic lesions	Infantile spasms symptomatic of acute asphyxia	16	1–2 s	25 (6–80)	Typical hypsarythmia	High amplitude slow wave flattening of the ongoing activities
4	M	Vacuum delivery at 33 GA Ventricula septal defect operated without complication in the first month of life	Abnormal	3 months	No	Normal	Neurotransmitters pahology	17	1–2 s	38 (14–87)	Spikes and slow delta waves	Diffuse flattening concomitantly with the spasm movement
5	F	Birth by cesarean at 35 GA for IGUR	Abnormal	6 months	Myoclonic epileptic status began at 4 months	Normal	Mitochondrial cythopathy	10	1–2 s	26 (10–60)	No identifiable physiological organization, slow delta, theta activities superimposed with spikes	Slow waves activities and flattening of the ongoing activities
6	F	Eutocic delivery at term Meningoencephitis (streptococci B) in the first month	Abnormal	8 months	Partial clonic seizures	Large porencephalic cyst^a^	Infantile spasms symptomatic of meningoencephalitis in neonatal period	29	1–2 s	46 (14–100)	Asynchronous poor activity with theta low amplitude activities and spikes under Cz	Non typical changes on EEG activity

IGUR: Intra-Uterine Growth Retardation, GA: Gestational Age.

a cf. cerebral CT scan.

### 3.3 fNIRS data

Between 6 and 29 typical spasms (for patients #1 and #6, respectively) were recorded using simultaneous EEG/fNIRS. Each spasm lasted for 1 or 2 s, and the interval between spasms ranged from 6 to 110 s.

**Fig. 3:**
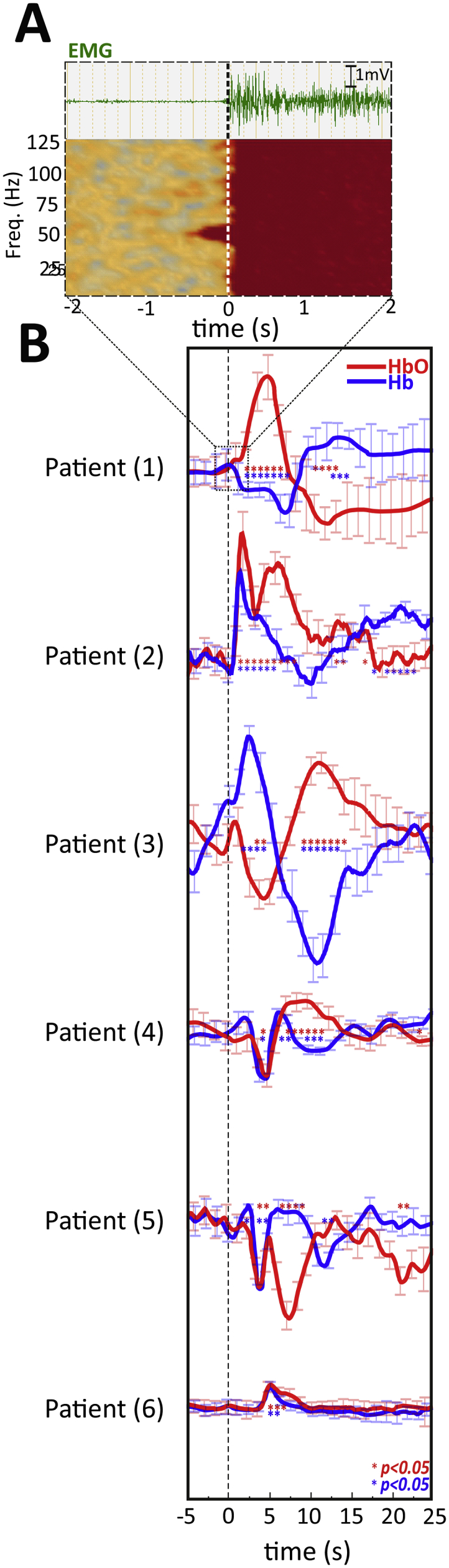
Spasm-related hemodynamics responses in the 6 patients A: a time-frequency response (TFR) of the deltoid EMG determined the onset of each spasm (T0). Spasm onset was always characterized by a sudden increase in the deltoid EMG power of all frequency bands between 0 and 100 Hz. B: In all patients other than the one with a large porencephalic cyst (patient #6), a two-phase hemodynamic change started with the onset of EMG activation (as determined in a time-frequency analysis). First, simultaneously with the spasm onset, a parallel shift on [HbO], [Hb] concentrations was observed, suggesting CBV changes; followed in all patients (other patient #6) by an opposite concomitant changes in [HbO] and [Hb], suggesting a NVC.

Combined EEG-multidistance fNIRS revealed changes in [HbO], [Hb] in all children (Fig. 3). In all children other than the one with a large porencephalic cyst, two hemodynamic phases were extracted from the optical signals. The first hemodynamic phase consisted of parallel changes in [HbO], [Hb], with a peak within 5 s of the onset of deltoid activity - suggesting that spasm was associated with initial changes in cerebral blood volume (CBV). In all patients other than the child with the large porencephalic cyst (patient #6), this was followed by NVC (i.e. concomitant opposite changes in [HbO] and [Hb]). The NVC was positive in 4 out of 5 patients (i.e. an increase in [HbO] and a decrease in [Hb]), with a peak 10 s after the peak in phase 1. The NVC was negative (i.e. a decrease in [HbO] and an increase in [Hb]) in the patient with a mitochondrial cytopathy (patient #5). In patient #6, only a small, late (5 s) but significant increase in both [HbO] and [Hb] was observed (Fig. 3).

#### 3.3.1 Cortical specificity of the hemodynamic response

**Fig. 4:**
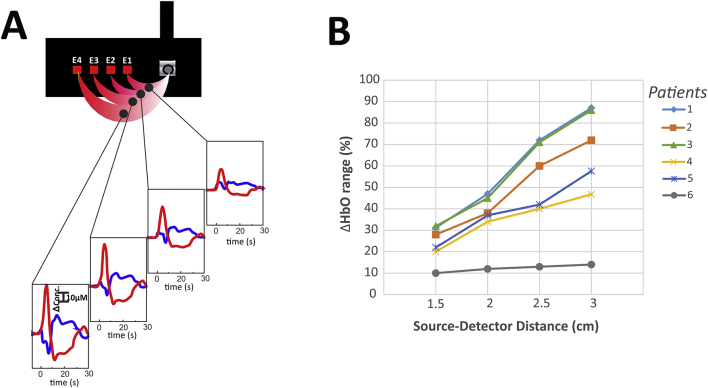
Multidistance NIRS analysis (patient #2) A: Hemodynamic changes observed with multidistance NIRS in the patient #1 B: Normalized range values of [HbO] for the four source-detector distances in the period of − 5 to 25 s vs source-detector distances (1.5, 2, 2.5, 3 cm) for the 6 patients. In all patients and whatever the hemodynamic phase, the changes in [HbO] and [Hb] increased with the emitter-detector distance – thus confirming the cortical origin of the hemodynamic changes.

To determine whether or not these hemodynamic changes had a cortical origin, we took advantage of the multidistance optical probe's ability to scan the optical changes at different tissue depths. In all patients and in both phases, the absolute amplitude of changes in [HbO] and [Hb] increased with the emitter-detector distance (Fig. 4A). The normalized [HbO] range increased with the source-detector distances increment (Fig. 4B).

This confirms the cortical origin of the hemodynamic response and suggests that the channels with the lowest emitter-detector distance (scanning superficial, non-cortical structures) are barely affected by the changes in chromophore concentration occurring in the deeper, cortical layers.

#### 3.3.2 The time course of the spasm-related hemodynamic response

To evaluate the time course of the spasm-related hemodynamic response, we analyzed simultaneous changes in the optical signal and TFR of the deltoid muscle activity (Fig. 3). Spasm onset was always characterized by a sudden increase in the deltoid EMG power in all frequency bands between 4 and 125 Hz (Fig. 3). In all patients other than the one with a large porencephalic cyst, the hemodynamic changes coincided with the onset of EMG activation. For the patient #1, hemodynamics changes [Hb] and [HbO] began 1.8 and 1.4 s before the onset of the contraction, respectively. We carefully checked the simultaneous video recording, but failed to observe any head or eye movements prior to the sudden onset of deltoid activity.

## 4 Discussion

In the present EEG-multidistance fNIRS study, we analyzed the hemodynamic changes related to each spasm in patients presenting clusters of infantile spasms. Although infantile spasms can be caused by a variety of disorders, the clinical and electroencephalographic phenotypes were stereotypical and thus suggesting the involvement of specific corticosubcortical processes and structures (Lado and Moshé, 2002). In this context, our study of a heterogeneous population evidenced stereotypical hemodynamic changes during infantile spasms, providing new arguments for a common pathophysiological substratum involving both subcortical and cortical structures.

### 4.1 Stereotypical hemodynamic changes characteristics

Hemodynamic changes comprised two distinct phases in all but one child (patient #6, with extensive cortical lesions).

In agreement with the literature (Haginoya et al., 2001, Haginoya et al., 2002, Haginoya et al., 2013, Munakata et al., 2004), the first phase was characterized by a rapid, parallel shift in [HbO], [Hb], which was suggestive of changes in CBV.

The second phase, observed in 5 patients (out of 6), was characterized by opposite changes in [HbO] and [Hb] concentrations within 6 to 10 s from the onset of spasms, consisted of a NVC (Buxton and Frank, 1997, Buxton et al., 2004). To the best of our knowledge, this is the first study to have demonstrated NVC during infantile spasms.

The involvement of NVC in infantile spasms fitted well with the classical balloon model (Buxton and Frank, 1997, Buxton et al., 2004) in at least 4 of the 6 patients. The coupling was positive in 4 cases and negative in 1 case. Interestingly, the only patient to display negative NVC was the one who was on a ketogenic diet due to a mitochondrial cytopathy, which results in changes in oxygen metabolism in the brain. In the present case, the mitochondrial cytopathy and the ketogenic diet might have biased the brain's energy towards an anaerobic metabolism (Danial et al., 2013). Buxton's model of NVC would not apply to this particular clinical situation. Our results may thus reflect a decrease in oxygen consumption due to primarily anaerobic metabolism. This hypothesis might help to understand the mechanisms underlying negative NVC but deserves further investigation.

### 4.2 The cortical origin of hemodynamic changes

To accurately describe the hemodynamic changes and notably determine their cortical origin, an EEG-multidistance fNIRS was used. Previous optical imaging studies used a probe with an emitter-detector distance of 25 mm (corresponding to channel A3 in the present study) (Munakata et al., 2004, Haginoya et al., 2002). This equates to a photon penetration of about 10 to 12 mm (Patterson et al., 1995). In our study, channels A1 and A2 (with an emitter-detector distance of 15 and 20 mm, respectively) were used to monitor systemic hemodynamic changes.

We did not observe any changes that solely affected these two channels. The changes in the concentration of the chromophores observed in channels A1 and A2 were always less pronounced than those observe in the channels with a greater emitter-detector distance (A3–A4), suggesting that the changes occurred in cortical structures and not in the skin as in the skull. These results support the idea that the origin of hemodynamics changes results from changes in the dynamic of cortical microvessels.

The absences of early CBV changes and the absence of NVC in the patient with a large porencephalic cyst (patient #6) might comfort the cortical origin of these hemodynamic changes and further support the idea that the origin are located within a functional cortical vascular network.

### 4.3 Local or diffuse cortical hemodynamic changes?

Hemodynamic signal have been recorded in all patients over frontal areas regardless of the etiology or of any brain damage.

In contrast to what was been previously hypothesized, notably in SPECT or fNIRS (Haginoya et al., 2001, Haginoya et al., 2002, Haginoya et al., 2013, Munakata et al., 2004, Asano et al., 2005, Nariai et al., 2011), our results suggest, that the vasomotor response is not a consequence of an increase in cortical focal activity linked to a cortical lesion.

To cover more globally the cortical hemodynamic evaluation, high density combined EEG-fNIRS should be used. Although our study focused in frontal area, the vasomotor responses is likely to be not restricted to this specific region and support the idea of a spatially diffuse cortical hemodynamic response. In the assumption that the two phases, CBV changes and NVC, arise in the cortical vascularization and appears to encompass a wide cortical area, our results support the hypothesis of a common trigger for each spasm inside the cluster of spasm not obligatory related to a cortical malformation which would interact with the activity of the entire brain. This does not preclude an effect on the hemodynamics of a focal cortical onset zone related to a focal cortical lesion (Asano et al., 2005, Nariai et al., 2011). The mechanisms that triggered and maintained the cluster of spasms are likely to be different from those involved in a spasm inside a series.

### 4.4 The involvement of cortical-subcortical areas in infantile spasms

Our results reinforce the hypothesis whereby a number of specific, complex processes in subcortical-cortical loops are involved in each spasm in a series of spasms (Lado and Moshé, 2002).

During the first hemodynamic phase, the simultaneous changes in CBV and motor activation likely results from the involvement of the brainstem. Subcortical structures have an important role in the control of cerebral blood flow and volume. The sympathetic nervous system, controlled by the brainstem, is likely to be involved in the adjustment of the CBV by modulating the contractility of both pericerebral vessels and deeper, intracerebral vessels (Langfitt and Kassell, 1968, Reis et al., 1984). In the same line, electrical and chemical stimulation of various brainstem structures elicits increases or decreases in the cortical CBV (Hamel, 2006). Therefore, our results support the hypothesis whereby pathological activation of the brainstem networks might be involved in these initial CBV changes.

However, CBV changes related to the cortical activation could not be definitively excluded. In some patients, initial CBV changes, which occurred simultaneously or prior to motor activation (patient #1) might be related to similar mechanisms that have been involved in the changes of the hemodynamic parameters that we previously described in absence seizures (Roche-Labarbe et al., 2010). Such early hemodynamic changes were likely to be related to the simultaneous changes in synchronization that occurred prior to the spike and wave discharges (Aarabi et al., 2008). One of the hypotheses of such early activity might be that NIRS rely on metabolic needs that are related to neuronal activation of synchronized and non-synchronized neurons whatever their orientations.

Nevertheless, infantile spasms could not be explained solely by cortical involvement. The occurrence of spasms without the two-phase hemodynamic changes in patients with very extensive cortical lesions (Haginoya et al., 1999) (e.g. patient #6 in the present study) or with hydranencephaly (Neuville, 1972), reinforces the “centrencephalic” theory (Kellaway et al., 1979, Lado and Moshé, 2002, Hrachovy and Frost, 1989). In parallel to its potential impact on the cortical vascular control, the activation of structures located in the brainstem triggers a pattern generator for spasms. This pattern generator like those involved in Moro reflex (Gobbi et al., 1987), startle reactions (Brown et al., 1991), bursts of sneezing and coughing spells (Wallois and Macron, 1994), trigger also coordinated clusters of movements, independently of any cortical circuit (Kellaway et al., 1979, Lado and Moshé, 2002, Hrachovy and Frost, 1989).

The latter second NVC phase suggests a secondary diffuse activation of the brain likely resulting too from the brainstem involvement (Lado and Moshé, 2002, Juhász et al., 2002) that triggers diffuse changes in cortical excitability, which, in turn, results in the diffuse electrodecremental event usually associated with fast rhythms on the electroencephalogram (Kellaway et al., 1979, Lado and Moshé, 2002, Jobe, 2002, Frost et al., 2011).

In this line, the observation of high frequency oscillations (HFO) with Stereotaxic-EEG in patients with focal cortical lesions is highly suggestive of neocortical involvement in the initiation of the cluster of spasms (Haginoya et al., 2013, Asano et al., 2005, Nariai et al., 2011).

## 5 Conclusion

Infantile spasms are a complex, epileptic manifestation that involves both cortical and subcortical structures. The existence of diffuse cortical NVC, related to an increase in brain metabolism during each infantile spasm, reflects the existence of diffuse cortical activation during each spasm. However, NVC was not observed in case of large porencephalic cyst, which suggests that the cortex is not involved in all infantile spasms. In fact, the NVC was preceded by a diffuse change in CBV - suggesting a possible initial involvement of the brainstem.

Infantile spasms are characterized by a complex, dynamic interplay between brain activity, blood volume and metabolism, with the involvement of cortical and subcortical structures. This interplay can be studied with simultaneous EEG-fNIRS monitoring.

## Funding

This research did not receive any specific grant from funding agencies in the public, commercial, or not-for-profit sectors.

## Authors contribution

Conceived and designed the experiments: Emilie BOUREL-PONCHEL, Mahdi MAHMOUDZAEDH, Fabrice WALLOIS.

Performed the experiments: Emilie BOUREL-PONCHEL, Mahdi MAHMOUDZAEDH, Aline DELIGNIERES.

Analyzed the data: Emilie BOUREL-PONCHEL, Mahdi MAHMOUDZAEDH, Fabrice WALLOIS.

Contributed reagents/materials/analysis tools: Emilie BOUREL-PONCHEL, Mahdi MAHMOUDZAEDH, Fabrice WALLOIS, Aline DELIGNIERES, Patrick BERQUIN.

Wrote the paper: Emilie BOUREL-PONCHEL, Mahdi MAHMOUDZAEDH, Fabrice WALLOIS.

Read and accepted the manuscript: Emilie BOUREL-PONCHEL, Mahdi MAHMOUDZAEDH, Fabrice WALLOIS. Aline DELIGNIERES, Patrick BERQUIN.

## Appendix A Supplementary data

Supplementary material Image 1
