# Supplementary material for: Non-invasive, multimodal analysis of cortical activity, blood volume and neurovascular coupling in infantile spasms using EEG-fNIRS monitoring
Source: Neuroimage Clin. 2017 May 13;15:359–66. doi: 10.1016/j.nicl.2017.05.004 (PMC5447509; doi:10.1016/j.nicl.2017.05.004)
Supplement: Supplementary file 1 — Supplementary material [file mmc1.docx]

**Supplementary material**

1. ***Data processing for EMG***

In order to determine the onset of EMG activity, a time-frequency representation (TFR) was generated by applying complex demodulation procedures (Hoechstetter at al., 2004; Papp and Harms, 1977) (for details, see supplemental data).

For each frequency of interest *ƒ_0_*, the following three steps were performed:

The original time-domain signal (i.e. with no offline filtering) was multiplied by *sin(2πƒ_0_ƭ)* and by *cos(2πƒ_0_ƭ)*. This modulation operation shifts every signal at frequency *ƒ* to the difference and sum frequencies (*ƒ± ƒ_0_*) in the frequency domain.

The two resulting signals were low-pass filtered to extract the frequency range originally centered around *ƒ_0_* and that had been shifted to the low frequency range (*ƒ- ƒ_0_*). Thus, the low-pass frequency cut-off corresponds to half of the width of the frequency band for which the envelope amplitude and phase are computed.

The output signals from step (ii) correspond to the real and imaginary parts of a complex signal over time. The magnitude of this complex signal corresponds to half the envelope amplitude.

Hence, to precisely determine the onset of EMG activity, a time-frequency analysis was performed for the frequencies between 4 and 125 Hz. Relative baseline segments lasting 1000 ms (from 2000 ms before the spams onset to 1000ms before onset) were defined for the EMG channel on which a seizure was marked. Frequencies were sampled in 2 Hz steps and latencies were sampled in 25 ms steps, corresponding to a TFR of ±2.83 Hz and ±39.4 ms in each time-frequency bin (full width at half-maximum). The TFRs of the EMG channels were expressed as the percentage power change in a time-frequency bin (relative to the mean power over the baseline epoch for that frequency): $TFR=\frac{P\left( t,f \right)-P_{\mathrm{baseline}}(f)}{P_{\mathrm{baseline}}(f)}. 100\%$ where *P(t,ƒ)* is the power at time *t* and frequency *ƒ* and $P_{\mathrm{baseline}}(f)$ = mean activity at frequency *ƒ* over the baseline epoch.

1. **EEG and clinical details for each patients**

The patients’ clinical characteristics, interictal EEG data and ictal EEG data are summarized in Table 1.

*Patient #1* presented with idiopathic infantile spasms. The spasms were first reported at the age of 6 weeks and were accompanied by developmental regression. The brain MRI appeared to be normal. After 6 months of standard treatment (vigabatrin and hydrocortisone), the clinical outcome was good. Interictal EEG revealed bilateral, high-amplitude posterior delta waves but no hypsarrhythmia. The infantile spasms (usually symmetric, with muscle flexion) occurred in clusters and were generally (although not always) associated with widespread theta activity on the electroencephalogram.

*Patient #2* presented symptomatic infantile spasms as the result of left temporo-parietal polymicrogyria. The first spasms were reported at the age of 5 months and had been preceded (at 3 months) by partial clonic seizures. The spasms were treatment-refractory. Asymmetric hypsarrhythmia in the left hemisphere was observed on the interictal electroencephalogram. The infantile spasms were symmetric. The ictal electroencephalogram was characterized by symmetric, high-amplitude slow waves and attenuation of the background activity.

*Patient #3* was delivered by emergency cesarean section at 40 weeks of gestation, following the observation of acute fetal distress syndrome and perinatal anoxo-ischemia. Clusters of infantile spasms were first observed at the age of 5 months and featured symmetric muscle flexion, psychomotor regression and hypsarrhythmia on EEG. The clinical outcome was characterized by delayed psychomotor development, refractory epileptic encephalopathy and the persistence of spasms.

*Patient #4* presented with symptomatic infantile spasms as a result of a neurotransmitter deficiency (suggested by a very low level of 5-methyltetrahydrofolate in the cerebrospinal fluid). The first spasms were observed with EEG at the age of 3 months. The patient’s clinical status was characterized by psychomotor retardation, refractory epileptic encephalopathy, the persistence of spasms and various non-epileptic movement disorders. On the interictal electroencephalogram, a disorganized pattern was observed with spikes and slow delta waves. Spasms were characterized by symmetric bending movements of the neck and trunk, rolling of the eye and (in some instances) a concomitant slow wave and then an attenuation of the background activity on EEG.

*Patient #5* was delivered by cesarean section after 35 weeks of gestation, following the observation of intra-uterine growth retardation. Epilepsy started at the age of four months, with erratic cortical and subcortical myoclonus. The infantile spasms first occurred at the age of six months. A mitochondrial cytopathy was diagnosed, with a confirmed lack of complex IV in muscle and liver biopsies. The child developed severe epileptic encephalopathy and psychomotor retardation, and died from respiratory failure at the age of 18 months.

Standard EEG revealed highly perturbed background activity, a lack of identifiable physiological features and abnormally high levels of slow theta/delta activity. The spasms featured muscle flexion on both sides of the body but mostly affected the left upper limb. Ictal EEG revealed a spike preceding muscle contraction, a slow wave and then an attenuation of the background activity.

The last patient *(Patient #6)* presented with Group B *Streptococcus* meningoencephalitis at the age of 1 month. The infection had caused severe anoxic and ischemic damage. A CT scan revealed almost complete destruction of the parenchyma and a thin cortical ribbon resulting in a very large porencephalic cyst (Figure 2). Symptomatic infantile spasms were first observed at the age of 8 months. The clinical outcome 24 months after the initial episode was characterized by refractory epilepsy and psychomotor retardation. In the interictal period, the EEG was asynchronous with low-amplitude theta and delta activities, and spikes on Cz only without change during cluster of spasms.
